# Listen to the Children: Elementary School Students' Perspectives on a Mindfulness Intervention

**DOI:** 10.1007/s10826-022-02292-3

**Published:** 2022-04-29

**Authors:** Andrea M. D’Alessandro, Kaitlyn M. Butterfield, Lerna Hanceroglu, Kim P. Roberts

**Affiliations:** 1 McMaster University, Hamilton, ON Canada; 2 York University, Toronto, ON Canada; 3 University of Windsor, Windsor, ON Canada; 4 Wilfrid Laurier University, 75 University Ave West, Waterloo, ON N2L 3C5 Canada

**Keywords:** Mindfulness, Student perspectives, Semi-structured interviews, Classroom-based mindfulness intervention, Self-regulation

## Abstract

In recent years, mindfulness-based practices in grade schools have been associated with students’ improved cognitive skills and general classroom behavior. In the majority of studies, however, only teacher and parent feedback are elicited, omitting a considerably significant voice – that of the students. Our study aims to fill this gap by exploring student opinions and perceptions regarding the implementation of a classroom-based mindfulness program. Elementary school students (*N* = 51) took part in teacher-facilitated mindfulness activities which were incorporated into their daily classroom routines. Over the course of the 8-week intervention period, students participated in focus groups about their perceptions of the program. Through qualitative content analysis, two major findings emerged from the focus group data: student opinions about the mindfulness program varied substantially and the mindfulness activities were not always liked and enjoyed. Critically, if students do not enjoy classroom-based mindfulness programs, they may be less motivated to engage in mindful activities and in turn may not experience the benefits that mindfulness has to offer. To maximize student engagement with mindfulness while addressing their concerns, the following recommendations are made: A balance between the entertaining and educational aspects of the program, flexible program delivery, and encouraging students to pursue mindful living outside of the classroom. This research is important to educational and clinical practitioners as student insight will benefit the development and modification of classroom-based mindfulness programs to ensure that students are better able to engage with and benefit from these programs.

## Highlights

- This study investigated 6th-and-8th-grade students’ experience with an 8-week classroom-based mindfulness program.
- Focus groups were held to gain a comprehensive understanding of children’s perspectives on the mindfulness program.
- Qualitative content analysis of the focus group data revealed key themes from the children’s narratives.
- Students opinions varied and the mindfulness activities were not always enjoyed.
- Balance, flexibility, and encouragement are recommended to maximize student engagement with mindfulness activities.

Mindfulness-based interventions have been of considerable interest to school psychologists, teachers and parents over the past decade given empirical evidence of the beneficial effects of mindfulness on children’s attention, well-being, self-regulation and cognitive control (Schonert-Reichl et al., 2015). The majority of published studies highlight parent and teacher perspectives of classroom-based mindfulness interventions (e.g., Layland, 2019, Schonert-Reichl & Lawlor, 2010) with quantitative analyses of children’s cognitive and emotional outcomes post-intervention (e.g., Flook et al., 2010; Schonert-Reichl et al., 2015). First-hand accounts from the children who participate in mindfulness interventions, however, are rarely presented (Sapthiang et al., 2019). It is imperative to investigate children’s actual experience with mindful programming to ensure that they are comfortable engaging in mindful activities and are able to experience the benefits of mindfulness training in their future lives. Additionally, student opinions will provide necessary data to continually raise the quality of mindful programming in schools.

## Benefits of Classroom-Based Mindfulness

Jon Kabat-Zinn (2003) defined mindfulness as the practice of purposefully orienting attention to the present internal and external environment while embracing experience nonjudgmentally. The origins of mindful practice rest in traditional Buddhist meditation that emphasizes awareness that emerges through paying attention to the present moment through the acceptance of moment-by-moment experiences in a nonjudgmental manner (Kabat-Zinn, 2003). Westernized mindfulness programs take a more secular approach where the purposive, nonjudgmental direction of attention to the present moment is applied both through meditation (as in traditional approaches) and less traditional activities such as eating or listening (Albrecht et al., 2012). Mindfulness training is effective in improving physical and emotional health in adults (Teasdale, 1999) and has thus been applied to numerous clinical and non-clinical domains including, but not limited to, chronic pain management (e.g., Kabat-Zinn, 1982; Moore & Martin, 2015), attention deficit hyperactivity disorder (e.g., van der Oord et al., 2012; van der Weijer-Bergsma et al., 2012), anxiety (e.g., Koszycki et al., 2007; Craigie et al., 2008), and depression (e.g., Ramel et al., 2004; Finucane & Mercer, 2006).

More recently, mindfulness-based practices have been implemented within grade school classrooms worldwide to target self-regulation, attention, and general classroom behavior (Black & Fernando, 2014, Malboeuf-Hurtubise et al., 2017, Napoli et al., 2005, Sapthiang et al., 2019, Sciutto et al., 2021, Willis & Dinehart, 2014). Mindful programs generally result in positive impacts on school-aged children within the domains of academic achievement (e.g., through improved attentional control) (e.g., Bakosh et al., 2016, Hanceroglu, 2017, Shoval, 2011), social and emotional well-being (e.g., Schonert-Reichl & Lawlor, 2010, Semple et al., 2010, Wall, 2005) and improvements in both symptoms of internalizing and externalizing disorders (e.g., Bögels et al., 2008, Felver et al., 2013, Malboeuf-Hurtubise et al., 2017, Napoli et al., 2005, Sciutto et al., 2021, Singh et al., 2010). For instance, Bakosh et al. (2016) investigated the feasibility and effectiveness of an 8-week mindfulness-based social and emotional learning program on the academic performance of third graders. The teacher-delivered mindful programming involved 10 min per day of a pre-recorded audio guide based on mindfulness-based stress reduction practices that encourage awareness of the present internal and external environment. Compared to controls, students who participated in the mindful programming achieved significant enhancements in quarterly grades for both reading and science.

In another study, a randomized control design was used to compare the effectiveness of a mindfulness program (MindUp!) to a social responsibility control program implemented by classroom teachers (Schonert-Reichl et al., 2015). Using a pre-and-post-intervention design, a range of cognitive-behavioral, neurophysiological, and psychological measures were collected from 4th-and 5th-grade students. After the 12-weeks of the programs, students assigned to the mindfulness program demonstrated improvements in cognitive control, stress levels, empathy, optimism, depressive symptoms, aggression, and prosociality compared to their counterparts in the social responsibility program. Finally, Malboeuf-Hurtubise et al. (2017) investigated the effects of a classroom-based mindfulness program among students aged 9 to 12 years old with learning disabilities. Problem behaviors (e.g., inattention, conduct problems) and symptoms of anxiety and depression among the sample were assessed with a pre-and-post-test design. Here, the 8-week cognitive-therapy-based mindfulness program was associated with reduced frequency of target symptoms in the students (Malboeuf-Hurtubise et al., 2017).

Parent and teacher perspectives of classroom-based mindfulness programming are generally positive given the academic, social, and emotional benefits to children (Black & Fernando, 2014, Bögels et al., 2008, Wall, 2005). Importantly, teachers’ narrative responses about classroom-based mindfulness interventions acknowledge widespread variation in students’ experiences with mindful programming. For instance, one qualitative study evaluated a mindfulness program 1 year after its incorporation into three elementary schools for children aged 7 to 12 years old (Campion & Rocco, 2009). The semi-structured interviews with 54 students, 7 parents and 19 teachers revealed a positive perception of program benefits. The results of the interviews with students revealed calmness, stress management and improved concentration as positive aspects of mindfulness, with boredom and tiredness cited as negative aspects of the mindful programming. Parents regarded the long-term benefits of mindfulness, particularly stress management, to be valuable as their children faced increasing pressures. Teachers noted the improvement in general classroom behavior and student appreciation of the program, yet almost a third of the teachers interviewed reported student negativity toward meditation, and 16% of teachers noted student resistance to the program (Campion & Rocco, 2009). Thus, while mindfulness was perceived to be beneficial for students by both parents and teachers, teachers noted that students held mixed attitudes toward the mindful activities.

## Children’s Opinions on Classroom-Based Mindfulness

Limited research has considered children’s perspectives of classroom-based mindfulness interventions (e.g., Ager et al., 2015, Campion & Rocco, 2009, Cruchon, 2009, Dariotis et al., 2017, Sapthiang et al., 2019, Thomas & Atkinson, 2017). For instance, Thomas and Atkinson (2017) investigated student and teacher perceptions of a classroom-based mindfulness program (Paws.b) that was implemented independently by the school and administered via a trained mindfulness teacher. The sample of 8-and-9-year-old students discussed their experience with the mindfulness program during post-intervention focus groups. The students expressed initial interest in the program which grew as they learned more about mindfulness. The students were grateful for the opportunity to participate in a mindfulness program and particularly enjoyed learning about the brain. Improvements in student attention, self-regulation, and relaxation were documented. The teacher-student relationships in the classroom were also reported to benefit from Paws.b. Importantly, the benefits of mindfulness training were reported to extend from the classroom to the students’ home life (Thomas & Atkinson, 2017).

Ager et al., 2015 qualitative study focused solely on student perspectives of a mindfulness program that was delivered by the school counselor and well-being director. Eighteen 6-and-7-year-old and twenty 9-and-10-year-old students completed a 10-week mindfulness program called Meditation Capsules and kept a mindfulness journal to document their perceptions of the program. Thematic analysis of the students’ mindfulness journals revealed that they perceived positive outcomes from the program. Students described an increased awareness of happiness, calmness, and peace and believed that mindfulness could be used to manage difficult emotions such as stress and anger. The students wrote of increased awareness of themselves, others and the environment, and expressed that mindfulness would be effective to deal with conflicts with siblings and friends. Altogether, students in this study perceived mindfulness to yield holistic benefits to their mind, body, and emotions (Ager et al., 2015).

## The Current Study

Of the limited research investigating student perceptions of mindfulness programming, most studies are outcome-focused. While understanding the cognitive, social and emotional implications of classroom-based mindfulness programs is necessary, this type of quantitative data are not sufficient to understand if children are able to use and benefit from mindfulness in the way that they are intended to. This issue is further impacted as the voices of parents and teachers have received more attention than that of the children participating in mindfulness programs. Critically, in a recent systematic review of qualitative studies investigating students’ experiences with mindfulness in schools, Sapthiang et al. (2019) reported that only seven studies met their inclusion criteria. Understanding children’s perspectives on classroom-based mindfulness programming can only serve to improve these programs which are intended to benefit children themselves. Indeed, in an investigation of factors related to the successful implementation of a classroom-based mindfulness program, Dariotis et al. (2017) reported that students and teachers had specific opinions about delivery of the program, including environment and time of day, as well as about the qualities of the mindfulness instructor. Here, Dariotis et al. (2017) highlighted that eliciting feedback from teachers and students will continue to be essential in understanding best practices to implement, adapt and develop classroom-based mindfulness programs. Thus, the purpose of our study was to address the shortcomings in the literature surrounding classroom-based mindfulness interventions and consider student perspectives on mindful programming through a qualitative analysis. Fifty-one elementary school students participated in an 8-week mindfulness program woven into the classroom schedule and conducted by the classroom teacher. We facilitated focus groups throughout the 8-week intervention period to explore student opinions and perceptions of the program. By eliciting children’s reaction to the mindful activities, we obtained first-hand accounts of their experiences that will serve to advance and modify mindfulness programs for children.

## Method

### Participants

Fifty-one students from a 6th-grade (*n* = 18) and two 8th-grade (8A; *n* = 18, 8B; *n* = 15) classrooms from the same school participated in this study. A second focus group of grade 6 students was conducted, but their data was inaccessible at the time of data analysis due to COVID-19 lockdowns and is therefore left out of this paper. Participants ranged in age from 11 to 14 years (*M* = 12.94, *SD* = 0.99) and included 28 (55%) female students and 23 (45%) male students. The participants identified as 56.8% White, 37.3% Asian or Pacific Islander; and 5.9% declined to indicate their ethnicity.

Written consent to participate and audio-record the focus group sessions was obtained from parents of student participants, in addition to oral assent from students, prior to commencing the study. Students were told explicitly that their responses to the focus group questions were being recorded prior to initiating each of the focus group sessions and that personal identifying information would not be linked to their responses. Students were not required to respond to all focus group questions and, thus, students who raised their hands to answer the focus group questions were selected and audio-recorded. Some parents additionally agreed to have their children’s responses quoted for publication and/or presentations without having any personal identifiable information linked to their responses by checking a box in the consent form. Ethics approval was obtained by the University Research Ethics Board at [name of university] (REB#4833). The project was also reviewed and approved by the Research Review Committee of the regional school board.

### Procedure

#### The classroom-based mindfulness program

The mindfulness program was intermittently taught to teachers by one of the authors (L.H.). Before beginning the program, teachers were trained on two mindful activities and were asked to incorporate them into their classrooms for the following 2 weeks (Week 1 and Week 2). During Week 3, research assistants observed the classroom, held a focus group interview with students, and trained teachers on two more mindful activities to incorporate into the following 2 weeks (Week 4 and Week 5). This cycle continued through to Week 8. Mindful activities within the current study followed the MindfulMe! program, which was adapted from resources from mindfulness organizations on the web and the MindUP! program (The Hawn Foundation, 2011; see Hanceroglu, 2017 for more information on the creation of the MindfulMe! curriculum). This program included a range of mindfulness-based activities (see Table 1 for a list of the weekly activity schedule). Students engaged in the program during the school day in their regular classrooms.

**Table 1:** MindfulMe! program activities

Week	Activity	Mindfulness aspect
1	Relaxation	Students learn mindful breathing techniques and how to complete a body scan.
2	Mindful movements	Students practice being aware of body sensations, recognizing physical sensations both when at rest and active and stretching (yoga) to focus on body strength.
3	Self-compassion (letting go of anger)	Students learn self-compassion and letting go of negative emotions. Students drop a shell into a jar filled with water and imagine their anger settling from the head to the heart as the shell settles in the jar.
4	Gratitude (thankfulness)	Students learn to cultivate calm and comfort when expressing thankfulness and joy by decorating and filling a mind map with the people they are thankful for.
5	Mindful listening	Students learn to focus and be responsive to sounds in the environment (e.g., rainforest soundtrack) by recording what they hear and sharing their responses with their classmates.
6	Worry	Students focus on breathing and rejecting anxious thoughts by shaking glitter bottles (containing liquid and suspended glitter) and focusing on the glitter settling.
7	Mindful eating	Students learn to eat slowly and notice the flavor and temperature of the food by describing their sensory experiences and facilitating mindful awareness of the senses.
8	Spiderman	Students engage in a compilation of the mindful movements and mindful listening activities.

#### Focus groups

Students participated in researcher-facilitated focus groups with their classmates on four occasions throughout the 8-week mindfulness intervention. A semi-structured interview format was used in focus group sessions lasting approximately 30 min. Students were asked questions related to the following research questions (see Table 2 for the focus group questions):

1. What were the students’ opinions on the implementation of the MindfulMe! curriculum?
2. What were the students’ perceptions of mindfulness prior to experiencing the MindfulMe! curriculum compared to after completing the 8-week mindfulness intervention?
3. What impact did the students recognize both personally and within the classroom as a whole after completing the 8-week mindfulness intervention?

**Table 2:** Focus group questions

Theme	Question
Perceptions of mindfulness	What does mindfulness mean to you?
	Who has heard of mindfulness before?
	Have your thoughts about mindfulness changed since the beginning of the program?
	Are you a supporter of mindfulness (after the program)?
Program implementation	What do you like about any of the mindfulness exercises being delivered by your teacher?
	What do you not like about any of the mindfulness exercises being delivered by your teacher?
	What are your suggestions about changing any of the exercises?
	Would you recommend that this mindfulness program be implemented in other schools?
Program outcomes	Has anyone been practicing the mindful activities outside of class?
	Have any of the mindfulness exercises been helping you?
	Is the class atmosphere any different after completing one of the mindful activities compared to other classes when we do not engage in an activity?

Students were asked these questions only at the focus group sessions, which occurred at three different time points.

### Data Analysis

The focus group discussions were audio-recorded and transcribed verbatim. The transcribed data from the three classrooms were amalgamated and organized into a response chart according to interview question, yielding 453 student responses to the focus group questions to be considered in data analysis. Excel software was used for the qualitative thematic analysis via a data-driven approach where emergent themes and patterns were drawn from the data. Thematic analysis followed Braun and Clarke’s (2006) guidelines. Here, three independent coders (the first author and two trained research assistants) first familiarized themselves with the data, then generated initial codes by addressing every response provided. The coders’ independent thematic coding charts were compared and discrepancies between themes were discussed and resolved. From this analysis, agreed-upon themes were defined and those which were judged to be repetitive themes were amalgamated and redefined. A second round of coding was completed by the same three original coders where codes were analyzed and combined as deemed necessary. Once again, the coders’ independent thematic coding charts were compared and discrepancies in the application of themes to the children’s responses were discussed and resolved. At this stage, 49 themes were identified. The first author collated the 49 themes into six key themes that were discussed and approved by the second author. For a random 20% sample of the responses (90 responses), Cohen’s Kappa revealed moderate agreement (κ = 72.05%). Discrepancies between the coders’ work were discussed to arrive at full agreement for the 20% sample. Themes were then updated and redefined to better reflect the data.

## Results

The data provide a cohesive picture of the students’ general perception of mindfulness and their opinions specifically relating to the MindfulMe! program. Emergent themes from the data set included emotional and behavioral regulation, barriers, delivery, applications, personal and social awareness, and enjoyment. Descriptions and examples of the themes are provided in Table 3.

**Table 3:** Themes, descriptions, and examples

Themes	Description	Example responses
Emotional and behavioral regulation	Students noticed changes in emotions, energy levels and behavior, both at an individual and classroom level in relation to the mindfulness program. Specifically, students noted a calm and quiet atmosphere, decreased hyperactivity, reduced stress, reduced arguments, as well as increased energy and organization.	“I think we are a bit more calmer after the body scan.” (6th-grade student) “I like the body scan because when you get out of it you feel like you just had woke up from the best nap.” (6th-grade student) “I feel like the stretching, uh after we are done, I stop like fidgeting with things.” (6th-grade student)
Barriers	Students mentioned several barriers to their ability to enjoy and benefit from the mindfulness program. Students described being distracted and uncomfortable and at times perceiving activities to be pointless. Students mentioned that some activities were difficult, made them tired, brought about negative emotions, or were not perceived to be age appropriate. Students explained how the variable outcomes associated with mindfulness impacted their perspectives.	“…like when I come, I’m like ready to start right away with the work but like after we do like the mindful, like uh the body scan or listening like I kind of like lose interest – lose focus in my work.” (8th-grade student) “I didn’t like when we did the mindful body scan, when our eyes were closed cause when I keep my eyes closed, I feel like I get distracted more.” (8th-grade student) “Whenever I close my eyes to do anything, I zone out and get really tired and wanna sleep. So, uh, sometimes when I’m closing my eyes during the body scan, I sort of don’t hear what they are saying. I just sort of drift and sleep.” (6th-grade student)
Delivery	Students had varied opinions about the delivery of the mindfulness program. Some students felt restricted by the instructions for various activities. Students desired flexibility in choosing activities and a variety of activities to choose from. The environment of the activities were important to the children, as well as the time of day, the length of the activity, the consistency of the program and the familiarity the students had with the mindfulness leader.	“I liked for listening when we had the option of which sounds to listen to, and we would have different ones.” (8th-grade student) “I think that we should make them a little bit longer because 5 min doesn’t really give a lot of time.” (8th-grade student) “A lot of these activities are more like auditory based or visual. I kind of want like a hands-on activity, like popping bubble wrap.” (8th-grade student)
Applications	The students described applying the learned mindfulness techniques in a range of circumstances, including their daily routines, in dealing with difficult emotions, falling asleep, sports and homework.	“Usually, when I have mixed emotions about something, or I’m in a certain mood, it’s kind of hard for me to think straight and like focus on one thing at a time because I’m always thinking about everything at the same time. So, that way I can now do the breathing exercises. I can focus on things.” (6th-grade student) “I’ve done it once or twice before my hockey games, in the car. So, it gives me focus.” (8th-grade student) “I feel like breathing, like mindfully breathing, before going to bed actually gives me a more relaxing sleep.” (6th-grade student)
Personal and Social Awareness	The children perceived a major benefit of mindfulness to be increased focused. Students discussed an increased awareness of internal states, as well as the ability to mentally distance themselves from the external environment. Students also reported an increased ability to introspect and consider the experiences of others.	“I found that when I was listening to it, I kind of found that part, what I was hearing… I was more aware of what was going on.” (8th-grade student) “When I mindfully listen, it kind of feels like I’m in my own world.” (6th-grade student) “I like the body scan because it really relaxes me, and I can focus on what we are going to do next in school.” (6th-grade student)
Enjoyment	Students enjoyed many aspects of the mindfulness program. The community atmosphere when the whole class gathered to participate in the activities was greatly enjoyed. The students also had fun during the program, found the activities helpful, enjoyed the esthetics of the activity materials and were comfortable.	“I think we should continue the mindfulness scan. It’s very relaxing and it’s nice to listen to different sounds in the classroom together.” (8th-grade student) “Uh yeah, I think it was pretty relaxing and challenging to do the yoga, yeah, it was fun.” (8th-grade student) “When we first started, I didn’t know what mindfulness was but now after 4 months, I think it was really helpful for me and I will use it in the future.” (8th-grade student)

### Emotional and Behavioral Regulation

Students discussed perceived outcomes of mindfulness related to personal and classroom regulation. For instance, many students were in favor of continuing mindfulness due to the calming and relaxing outcomes of the activities. An 8th-grade student who particularly enjoyed the mindful listening activity said: “…[I enjoyed the] mindful listening because I find it the most um, personally, the most calming and soothing and it makes me feel good.” Several students described how the mindfulness activities, especially the body scan, left them feeling rejuvenated. One 8th-grade student put it this way: “Like for me, like the body scan, um, once we get to core usually it’s like later, and after doing all the work my brain is like tired and then once we do the body scan it’s like my brain is better.” Students also noted behavioral changes related to mindfulness, with a 6th-grade student highlighting that they enjoyed the body scan activity “…because it helps me be less hyper throughout the day.”

Students perceived that the mindfulness activities regulated the classroom atmosphere: “I noticed that after we do mindfulness activities, um, I notice the atmosphere is a little quieter,” (6th-grade student) and believed the class to be “a bit more organized” on days that the mindfulness activities were completed compared to days in which the activities were not completed (6th-grade student). Furthermore, the students perceived a direct connection between the mindfulness activities and class arguments: “When we started doing the mindfulness activities at break, our class like didn’t fight as much as we used to. Then as soon as we stopped doing them as much, everybody started fighting over every single thing,” (6th-grade student).

### Barriers

The students described a broad range of barriers to their engagement and enjoyment of the mindfulness activities. Some students reported difficulty concentrating as a key barrier: “It is hard to focus sometimes on the body scan - a bunch of people here laughing, and I’m usually more focused on who’s laughing or something” (8th-grade student). Some students shared that the activities made them more tired than they were when they got to school:

“When I go to school in the morning, I feel already really exhausted and then when we do the body scan in the morning, I feel like I just want to go back and sleep for the rest of the day and when I feel really, really tired, I don’t feel like doing things,” (8th-grade student).

A few students expressed that they felt the activities were boring (e.g., “I don’t really want to continue. I don’t like it because it’s boring,” – 8th-grade student). Some students felt uncomfortable during some mindful activities, such as one student who noted:

“I don’t like the butterfly pose either because even though you ask us not to pull your legs down, sometimes when gravity pulls down my leg, it hurts and strains right here, but then it’s tiring so I have to put it down and then it hurts again,” (6th-grade student).

Students felt that some aspects of the program where pointless and this contributed to their dislike of the program. For instance, an 8th-grade student had a hard time understanding the intention behind the body scan: “I don’t like the body scan because – maybe I think it’s the wording of the script. Like, I guess it’s like ‘feel your legs and the tension,’ so you’re bound to feel tension ‘cause you are looking for the tension.” The students found certain activities, such as mindful breathing, to be difficult, where many agreed with this anecdote: “The breathing one we did today, we had to breathe in for 6 s and then hold it for 6 s and then breathe back out for 6 s. It’s sort of hard because I can’t even breathe in for 6 s,” (6th-grade student). When reflecting on the letting go of anger exercise, an 8th-grade student shared that this activity made her anxious: “Okay, so the shell jar, when I thought about it, I got more anxious because I thought I didn’t have anything I was angry about, but then it just came back…” Another student shared of the sadness that came about when their shell jar did not work as planned, “I didn’t really like it cause when I put the shell in mine, it started floating and it didn’t sink and then my group was like ‘oh your anger is not gonna go away.’ It made me feel sad,” (8th-grade student).

A key issue for students that prevented them from enjoying the mindfulness activities was the perception of varied outcomes of mindfulness, both personally and within the classroom as a whole. For example, one student noted that the class atmosphere “is quieter on the day we do it right after the activity, but not in general,” (8th-grade student). An 8th-grade student said: “I don’t think we should really keep it cause it doesn’t affect me in any way, it just makes a little calmer but I just go back to how I was before.” These anecdotes demonstrate the students’ perceived variable nature of mindfulness when beneficial effects emerge from the program but only for a short time. Finally, when asked if they would recommend that mindfulness be implemented in other schools, a few students approved with the caveat that the programming be implemented for older students (e.g., “I feel like this would be better in high school cause they might be more stressed” – 8th-grade student).

### Delivery

Students’ had specific opinions about the delivery of the mindfulness program that varied greatly. The majority of students preferred that the mindful activities be delivered solely by their teacher due to familiarity, echoing the responses of an 8th-grade student who stated: “We want our teacher to do the talking instead of YouTube videos of strangers.” In contrast, one student shared:

“I really like how [teacher] like puts on YouTube videos of some girl saying it because when it’s her voice over and over it’s not as relaxing because we hear it every day. When it’s a different voice, um, it’s more relaxing because we don’t hear it as often,” (8th-grade student).

Students felt that the instructions of some of the activities were restrictive, especially closing one’s eyes, and desired to have the option to choose what to do. For example, an 8th-grade student said: “Well, um, at first I didn’t like how you kept your eyes open, but I suggested like, that we give kids the option to keep their eyes opened or closed, so I like that [teacher] gave us the option,” (8th-grade student). Related to a desire for variability and flexibility, many students were in agreement with one student who shared, “I actually really liked having the choice of which mindful activity we wanted to do because then we could choose which would benefit us the most,” (8th-grade student). The students made specific comments regarding the environment in which they engaged in mindfulness activities, with some particularly enjoying when the activities (e.g., mindful listening) were completed outside. Relatedly, an 8th-grade student was in favor of adding more sounds to the mindful experience: “…background noise would be helpful. Having the room like dead silent gets kind of awkward and uncomfortable for me…” The students also had specific and varied opinions about lighting, with some preferring the lights to be kept on and others preferring the natural light. Students were also divided in their opinions on the time of day that the mindfulness exercises be completed. An 8th-grade student shared:

“I feel like when we do it in the morning, it’s the beginning of the day, we’re already kind of useless when we are already calm. I find, like personally, I find that doing it in the morning makes me more tired. Because I’m adding like, it’s relaxing me even more which is kind of like shutting me down a bit,” (8th-grade student).

However, there was an equal number of student responses expressing a preference for mindfulness to be implemented into the morning routine, for example, an 8th-grade student shared: “I wish we had it like first-period cause then I’m like all tired and you just feel better.” Opposing opinions on duration were also illuminated, with some students feeling that 5 min activities were not long enough to fully engage in mindfulness and others arguing the opposite: “Personally, I think 5 min is too long. I feel like we should shorten the time. I can’t sit still for that long and then I get distracted.” The majority of students called for consistency in the frequency of the mindfulness programming in the classroom, yet an 8th-grade student opposed the majority’s call for consistency by saying:

“I feel like it helps me like to relax and stuff but it’s becoming like for me, I feel like it’s kind of annoying cause like it’s becoming more of a routine. So, like every time I come and like okay, we are doing the mindful scan or listening or whatever. So, I kind of like doing it like once in a while.”

### Applications

The students described using the mindfulness techniques learned in the classroom in a range of settings. Some students described incorporating mindfulness into their daily routines, including brushing their teeth, their morning routine (e.g., “When I wake up, I try and do a body scan as much as I can because it just helps me like wake up,” – 6th-grade student) or eating (e.g., “Before bedtime, I usually get a snack, so I just did the mindful eating,”- 6th-grade student). Students described using mindfulness in dealing with difficult emotions, such as the case with an 8th-grade student who shared: “The past weekend I had to do this thing in front of people, and I was really anxious, so I would just do the mindful scan to calm myself down and do the breathing. And before going up, I did the breathing to be calmer.” The gratitude log was used to deal with emotions as an 8th-grade student shared that “I’ve also used the thinking a lot more about gratitude. Um, especially if I’m agitated.” Relatedly, an 8th-grade student shared how the body scan helped calm them down at hockey: “Before hockey, my parents started really focusing on time and if we aren’t on time. I sometimes get really worked up, but now if it’s close, I get calmed down.” Another student described how mindful breathing during homework improved their success at arriving at the correct answers: “Like if you do the breathing exercise in the middle like if you’re having trouble, I find that it kind of helps me focus and helps me find the answers better,” (6th-grade student). Students also described using the mindfulness exercises, specifically the body scan, mindful movements, and spiderman activity, outside of the classroom for their physical benefits. A 6th-grade student shared “I like the exercises with the stretches, and you hold them because it relaxes you and it helps you with stretching and flexibility.” Several students shared that they practiced mindfulness to help them fall asleep at night: “Before, when I go to bed almost every single night, um, especially on those stressful days, I just like lay down in my bed for a few and do the body scan for like 5 min,” (6th-grade student). Finally, students discussed mindfulness was helpful when within the classroom (e.g., “Working independently in class,” (8th-grade student).

### Personal and Social Awareness

The students discussed an increased personal and social awareness related to the mindfulness activities. For instance, increased focus was perceived to be a major benefit of the mindfulness activities, where one student shared: “I think it has [helped] because it kind of gets you to zone in with your mind and it helps you focus on whatever you are doing,” (8th-grade student). The students also described increased awareness of internal states. For example, a 6th-grade student shared:

“I also like the body scan because when you have a lot of tension, you get a moment to like figure out how you feel because half of the day you’re all tensed up but then you can actually lay down for a few minutes and notice how you feel.”

The students discussed mindfulness allowing them to be immersed in thought to the point of separating oneself from the environment. This distancing was reflected in several responses in the focus group discussions, such as: “I also like the mindful listening. It’s nice to sort of just get lost in thought while also focusing at the same time if that’s the way I could describe it,” (8th-grade student). Several students shared that mindfulness led them to reflect on their personal experience and increase in gratitude. As an 8th-grade student put it, “I really like the gratitude because it makes us think about what we actually have. That we should be grateful for everything we do and not be selfish and think about what we don’t have,” and “…because it gets you to realize what you are grateful for because it’s not every day you realize what you have” (6th-grader). Students also expressed that they wanted to keep the gratitude log, emphasizing a comparison between their experience and that of others: “I wanna keep the gratitude one because I think we have it really good compared to people in other places and there is a lot to be grateful for, like even having the materials for class which we take for granted. I think if we really try, we could show – cherish how much we have,” (8th-grade student).

### Enjoyment

Students reported enjoying many aspects of the mindfulness program. One aspect that the students thoroughly enjoyed throughout was the community appeal of whole-class participation. An 8th-grade student shared:

“…I feel as though, as a class, because we are doing something together that we are all sharing that experience. Whether its mindfulness or not, just doing something together has been a positive – has had a positive effect on our community feel.”

The students described having fun during the mindfulness programming, specifically naming the letting go of anger, mindful movements, and gratitude log. The helpful aspect of the program was a reason that many students wanted to continue and recommended that mindfulness be implemented in other schools. For instance, an 8th-grade student shared “When we first started, I didn’t know what mindfulness was, but now after 4 months, I think it was really helpful for me and I will use it in the future.” Several students commented on the appealing nature of the aesthetics of activities (e.g., “I think the anger jars would still be useful in a classroom setting… It’s just satisfying to watch the shells go… I think they’re pretty” – 8th-grade student). Finally, some students reported that mindfulness increased their confidence and comfort. For example, an 8th-grade student said: “I like the body scan because after I come out of it, I feel more like, confident,” And a 6th-grader shared “I think when we do the stretches at school, um, it makes me feel more like comfortable at school I guess.”

## Discussion

Although mindfulness practice has been found to benefit children in a number of ways (Black & Fernando, 2014, Malboeuf-Hurtubise et al., 2017, Napoli et al., 2005, Sapthiang et al., 2019, Sciutto et al., 2021, Willis & Dinehart, 2014), research on children’s perspectives of mindfulness in the classroom are limited (Sapthiang et al., 2019). The present study thus adds to the paucity of literature on students’ opinions of mindfulness in the classroom and further speaks to children’s opinions on the implementation of these programs and their associated outcomes, which are critical for improving classroom-based mindfulness programs (Dariotis et al., 2017).

In their systematic review of qualitative research on children’s opinions of classroom-based mindfulness programs, Sapthiang et al. (2019) highlighted four major themes, including using attentional processes to regulate emotions and cognitions, stress reduction, improved coping and social skills and calming and/or relaxation. The results from the present study are consistent with such findings and build upon this literature by offering insight into how to improve mindfulness programs based on children’s opinions of delivery and barriers to their enjoyment and engagement with mindfulness. Here, our work reveals two major conclusions: first, students had widespread opinions on the program with individual children differing in their opinion often quite substantially; second, there was ample evidence that mindfulness activities were not always liked and enjoyed. Although disappointing, these findings are of critical importance as students who do not enjoy mindful activities may be less likely to engage in mindfulness and in turn may not experience the holistic benefits that mindfulness has to offer. Here, children’s explanations for their negative attitudes will allow practitioners to focus on the aspects that children thought ‘worked’ and avoid (or modify) the activities that children did not enjoy. While mindful practice is not designed to make a person happier, people are more likely to stick with programs that provide meaning and are enjoyable. Hence, if children enjoy mindful activities, they may be more likely to engage in mindful lifestyles after a program ends.

### Recommendations for Classroom-Based Mindfulness Practice

The findings have provided insight into children’s perspectives and have allowed for several recommendations for program development to be made. In order to capitalize on the aspects of the classroom-based mindfulness programming that the students enjoyed while addressing their areas of concern, the following recommendations are offered: A balance between the entertaining and educational aspects of the program, flexible program delivery, and encouraging students to pursue mindful living outside of the classroom (Table 4).

**Table 4:** Recommendations for classroom-based mindfulness practice

Recommendation	Description
Balance entertainment and education	To increase students’ appreciation for and engagement with mindfulness, the aspects of mindfulness that students enjoy should be maintained and built upon. Students may appreciate when the purpose of the activities and the reasoning for perceived restrictions are explained as this will provide them with insight into the benefits of mindful practice and the purpose of instructions that seem “pointless”
Flexible program delivery	Students have specific and varied opinions of program delivery. In response, the implementation of mindfulness should be based on day-to-day classroom observations at ideal, flexible times, rather than on a fixed schedule.
Encouraging mindful living outside of the classroom	Mindfulness curricula encourage mindful living outside of the classroom. This may be achieved by incorporating the application of mindfulness techniques into homework (e.g., at-home body scan for health class homework, or mindful listening as a homework task for a music class).

### Balancing Entertainment and Education

The majority of students in the present study indicated that they enjoyed the mindfulness program. When describing what they enjoyed about the program, the students listed whole-class participation in activities, the aesthetics of materials, the comfortable atmosphere, the helpful aspects of the activities and having fun as reasons for their enjoyment. The students also mentioned calmness and relaxation among other emotional and behavioral outcomes as reasons for their support of mindfulness. These enjoyed aspects are consistent with existing literature on students’ opinions on the benefits of mindfulness (e.g., Ager et al., 2015, Dariotis et al., 2017, Sapthiang et al., 2019)

With these positive attitudes, however, came several areas of dissatisfaction with the program. Some students felt that the activities were boring and that the mindful exercises were pointless. Some students shared that mindfulness would be better suited for high school students and many children were bothered by perceived restrictions, such as keeping one’s eyes closed or refraining from speaking to a classmate during certain activities. Although the children were dissatisfied with certain aspects of the program, the mindfulness scholar understands that the perceived restrictions or pointless elements are worthwhile and necessary for children to access the benefits of mindfulness training. For example, educators and clinical practitioners understand that closing one’s eyes and refraining from speaking to a friend during mindful practice are fundamental to the practice of focusing and cultivating an appreciation and awareness of the present moment. Furthermore, though students thought that the program may be better suited for older students with higher stress levels, educational and clinical practitioners see a benefit of learning mindfulness techniques in grades 6 and 8 as children of this age are at an important developmental stage where mindfulness may significantly impact executive functioning skills such as inhibitory control (Oberle et al., 2012) and equip them for dealing with future stressors (Taylor, 1996).

To maximize student enjoyment and engagement with mindfulness-based curricula and address their concerns with the programming, a balance is required between entertainment and education. The aspects of programming that students consider enjoyable should be built upon to draw students into the activities and increase their appreciation for the mindful program. Further, the purpose behind the activities and the reasoning for perceived restrictions should be explained to children to provide them with insight into the benefits of mindful practice in early adolescence and the purpose of instructions that seem “pointless”. For example, mindfulness programming could educate students on what executive functioning is and how mindfulness may improve this capacity. Educating students on this concept and on other benefits of mindfulness may be necessary for children to appreciate the perceivably restrictive instructions or pointless nature of activities as vital for the desired outcomes of mindfulness training. Increasing the educational aspects of classroom-based mindfulness programs is in keeping with Thomas and Atkinson’s (2017) research in which children specifically emphasized an appreciation for the educational aspect of learning about the brain mechanisms implicated in mindfulness. A balance between entertainment and education in mindfulness programming follows from the student perspectives shared in the present study and is suggested to ensure student engagement with and benefit from classroom-based mindfulness interventions. Another option might be to “phase in” mindful practices, for example, by beginning with a 2 min body scan which extends with practice to 5 min, and 10 min and so on.

### Flexible Program Delivery

The students described varied and specific opinions regarding program delivery. While topics such as the consistency, duration, environment and scheduling of the program were brought up in discussion, students were not necessarily in full agreement as to how the program could be changed to better suit both individual and classroom needs. The variation in children’s opinions on program delivery is consistent with Dariotis et al. (2017) findings where students and teachers had specific opinions about program delivery, including timing and environment. Further, the present findings are consistent with Campion and Rocco’s (2009) report of teachers observing variation in student reception to the mindfulness program.

Nevertheless, the who, what, when and where of the MindfulMe! program were important for the children and, thus, should be carefully considered in the development and implementation of classroom-based mindfulness programs. Student-focused mindfulness programming must address the delivery concerns highlighted by students in the present study to facilitate student engagement with mindfulness in the classroom. Here, the classroom teacher or mindfulness instructor should monitor students’ energy and attention levels throughout the day to identify when students might benefit most from the mindfulness activities. Based on the day-to-day classroom observations, mindfulness can be implemented into the classroom routine at ideal, flexible times, rather than on a fixed schedule and duration. Implementing a flexible program delivery schedule is recommended to address the children’s varying perceptions of program delivery and implement a student-focused aspect to mindful programming.

### Encouraging Mindful Living Outside of the Classroom

When asked if they had used any of the mindfulness activities at home, the students shared their experiences of engaging in various mindful techniques at home (e.g., brushing teeth, eating, dealing with negative emotions, completing homework, falling asleep). These results are encouraging as they demonstrate that students are willing and able to use mindfulness techniques outside of the classroom, yet contributions to the focus group discussion regarding mindfulness applications were quite low, yielding only 11% of the total (453) responses. One of the many possible explanations for poor participation with this question is a lack of student engagement with mindful practices outside of the classroom. As such, mindful curricula must place specific emphasis on the translation of mindful techniques from the classroom to the home and other spheres of life. Indeed, Sciutto et al. (2021) emphasized the necessity for rigorous inquiry of how students are engaged in mindfulness during and beyond classroom-based programming.

To encourage mindful living outside of the classroom, teachers could incorporate mindful techniques when assigning homework. For example, an at-home body scan might be part of the homework for health class or mindful listening as a homework task for a music class. As it is vital to ensure that students are comfortable and able to participate in mindfulness activities, encouraging students to cultivate a lifestyle of mindfulness will further increase this ability and likely produce the positive outcomes associated with mindful living.

Altogether, classroom teachers and mindfulness instructors should continue to utilize fun mindfulness activities that engage students in whole-class participation. Instructors should devote time in mindful practice to educate the students on the value of mindful living and encourage students to pursue mindfulness outside of the classroom. Finally, instructors should pay careful attention to the classroom on a day-to-day basis and deliver mindfulness training at flexible times when students may benefit the most.

### Limitations and Future Directions

The semi-structured focus groups in this study provided rich, anecdotal data on students’ experiences with the classroom-based mindfulness program MindfulMe!. However, these data must be interpreted with caution as the perspectives shared in focus group discussions may have been that of a limited number of the 51 students who participated in this study. Students were not required to contribute to the focus group discussions and the transcribed focus group data was de-identified for confidentiality reasons. Thus, it is possible that the responses shared in the present study came from the same few students rather than from the whole sample of 51 students. The present study should also be replicated with a methodology that ensures data collection from all participants similar to how Ager et al., (2015) used mindfulness journals to gather the perspective of each student. Furthermore, it is important to note that these findings are not generalizable to all age groups and thus developmental considerations must be made. Students who are older or younger than the grade 6 and 8 students in this study may have substantially different outlooks on mindfulness practices. Thus, future research should consider the perspectives of both older and younger cohorts in reference to classroom-based mindfulness programming. Finally, the present study used the classroom-based mindfulness program MindfulMe! that was inspired resources from mindfulness organizations on the web and the evidence-based MindUp! curriculum (The Hawn Foundation, 2011). As such, the findings of the present study may be particular to the MindfulMe! program (i.e., student perspectives on activities and associated materials) and may not translate entirely to other mindfulness programs. Nevertheless, the findings of the present study can be used as a general guide to further develop all classroom-based mindfulness programs. Despite these potential limitations, the present study provides important results adding to the paucity of qualitative studies investigating children’s perspectives on classroom-based mindfulness programming and yields specific areas to improve mindfulness programs based on children’s accounts.

## Conclusion

The present study investigated student perceptions of a classroom-based mindfulness program. Findings gleaned from the current study demonstrated substantial variation in student opinions about the mindfulness program and that the students did not always enjoy and like the activities. It is critical that educators and clinical practitioners pay attention to student opinions on mindfulness training as students who enjoy mindfulness are more likely to engage with the activities and in turn reap the holistic benefits that mindfulness has to offer. Based on student perceptions of the mindfulness activities, it is recommended that mindfulness programs for children ensure a balance between the entertaining and educational aspects of the program, allow for flexible program delivery and encourage students to cultivate a lifestyle of mindfulness outside of the classroom.

## Data Availability

The raw de-identified data can be requested from the corresponding author.
